# Appreciation of singing and speaking voices is highly idiosyncratic

**DOI:** 10.1098/rsos.241623

**Published:** 2024-11-06

**Authors:** Camila Bruder, Klaus Frieler, Pauline Larrouy-Maestri

**Affiliations:** ^1^ Max Planck Institute for Empirical Aesthetics, Frankfurt am Main, Germany; ^2^ Center for Language, Music, and Emotion (CLaME), New York, NY, USA

**Keywords:** aesthetics, liking, voice perception, voice attractiveness, vocalization

## Abstract

Voice preferences are an integral part of interpersonal interactions and shape how people connect with each other. While a large number of studies have investigated the mechanisms behind (speaking) voice attractiveness, very little research was dedicated to other types of vocalizations. In this Registered Report, we proposed to investigate voice preferences with an integrative approach. To this end, we used a newly recorded and validated stimulus set of contrasting vocalizations by 22 highly trained female singers speaking and singing the same material (in Brazilian Portuguese) in contrasting styles (sung as a lullaby, as a pop song or as an opera aria; and spoken aloud as if directed to an adult audience and as if directed to an infant). We asked 62 participants to rate these vocalizations in terms of how much they liked them; and we compared the amount of shared taste (that is, how much participants agreed in their preferences) across styles. We found highly idiosyncratic preferences across all styles. Our predictions concerning shared taste were not confirmed: although shared taste was higher for lullaby than for pop singing, it was unexpectedly higher for operatic than pop singing, and higher for infant-directed than adult-directed speech. Conversely, our prediction of limited consistency in average preferences for some singers across styles was confirmed, contradicting sexual selection-based ideas of singing and speaking as ‘backup’ signals of individual fitness. Our findings draw attention to the role of individual differences in voice preferences and highlight the need for a broader approach to understanding the underlying mechanisms of voice preferences. Stage 1 recommendation and review history: https://rr.peercommunityin.org/articles/rec?id=357. Stage 2 recommendation and review history: https://rr.peercommunityin.org/articles/rec?id=802.

## 1. Introduction

The voice is highly significant to human experience. Voice-selective areas have been described in the human cortex [1], and there is evidence for neural populations that respond selectively to songs [2]. Melodies are easier to remember when presented vocally than when played on a piano, banjo or marimba [3], even for trained pianists [4]. The voice is also incredibly flexible: it can serve a myriad of functions, and it sounds differently depending on its current use. Besides its obvious functions, that is, to express and exchange semantic meaning via speech, the voice conveys a wide range of non-verbal information. A person’s voice may cue the speakers’ body size and shape, health and age [5,6]. During speech, fluctuations in voice intonation (generally known as speech prosody or ‘the melody of speech’) may convey intent [7,8], emotional states [9–11], and even personality traits [12–14]. Across cultures, certain features consistently distinguish song and speech [15,16], and all studied cultures have some form of singing [17,18]. Both speech and song sound differently when directed to infants [19–21], and also in the case of singing, different uses of the voice are associated with different functions (e.g., loud, rhythmic singing in play songs to entertain, versus unaccompanied, soft and quiet singing of lullabies to soothe an infant [22,23]). Given the voice’s multiple facets, how can we understand individuals’ enjoyment of voices in different contexts? How shared are our preferences across different types of vocalization?

In the case of speaking voices, voice attractiveness is thought to signal the speaker’s physical fitness to potential mates. Voice attractiveness has been shown to covary with sexually dimorphic traits: individuals with more attractive voices also tend to have larger shoulder-to-hip ratios (for males) or smaller waist-to-hip ratios (for females) [24]. Voice attractiveness is also related to certain acoustic characteristics (e.g. higher fundamental frequency and more spread formants preferred for female’s voices, see [25]). More nuanced instances of voice attractiveness have also been described, with, for example, conformance to community speech norms increasing voice attractiveness ratings [26]. A different (though related) line of research has proposed a role for averageness and typicality in voice preferences. Bruckert *et al*. [27] found that morphed, averaged voices (which are smoother and have higher harmonics-to-noise ratios) were rated as more attractive than most of the individual voices presented to participants. Accordingly, average ratings of voice attractiveness have been reported to be highly correlated with ratings of stereotypicality [28], or negatively correlated with ratings of atypicality/distinctiveness in relation to an average voice [29]; though also see Mook & Mitchel [30] for a study where the positive effect of averageness (via morphing) on voice attractiveness was not replicated.

In the case of the singing voice, fewer studies have investigated the mechanisms behind our preferences. Bruder *et al*. [31] recently observed that participants’ ratings of 10 different perceptual vocal attributes (that is, articulation, breathiness, pitch accuracy, loudness, tempo, etc) were better predictors of listeners' liking of pop voices than computationally extracted acoustic features commonly used to describe voices such as jitter, shimmer, vibrato rate and extent and harmonics-to-noise ratio. Importantly, while preferences were highly idiosyncratic, as indicated by the low interrater agreement in liking ratings (Krippendorff’s *α* was 0.16), some average preferences emerged for some voices, as shown by highly correlated averaged liking ratings between the two experiments conducted (one with German and one with US participants). This suggests the emergence of robust average preferences amidst large individual differences in how participants perceive and like singing voices. Based on the literature on speaking voice attractiveness, it is difficult to say how much individual differences lie behind the typically reported average preferences. Valentova *et al*. [32] reported high correlations between average attractiveness ratings of spoken and sung vocalizations produced by the same subjects and argued that spoken and sung voices may work as ‘backup signals’ that convey the same information about a subject’s physical fitness. Because they used Cronbach’s *α* as a measure of interrater agreement, a reportedly problematic measure (it is inflated by larger sample sizes and ignores within-person variability [33,34]), the relationship between voice attractiveness of singing and speaking (as well as the role of individual differences in this relationship) needs to be clarified.

Here, we investigate the aesthetic appeal of a set of contrasting vocalizations—singing and speaking—in an integrative manner. We adopt an interactionist approach (e.g. [35]) within the larger framework of empirical aesthetics—an approach that takes into account aspects of the stimuli as well as subjective, internal factors related to the person making the aesthetic evaluation. This means that, in addition to examining mean liking ratings as an indication of average preferences, we also examine the variability in these ratings across participants.

**Figure 1.**
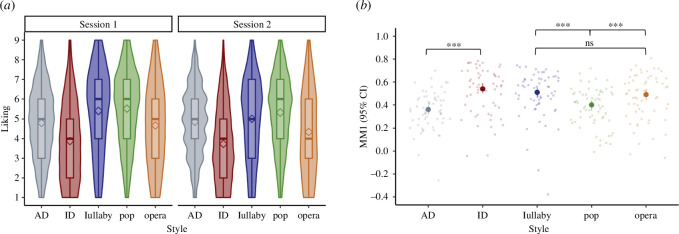
(*a*) Distribution of liking ratings by 62 participants (for each participant, *n* = 66 stimuli per style) in two testing sessions, by style of vocalization. Diamonds depict average liking ratings. Lower and upper hinges of boxplots correspond to the first and third quartiles, and whiskers extend from the hinge to 1.5 * inter-quartile range. (*b*) Interrater agreement or shared taste, as measured by ‘mean-minus-one’ agreement (MM1) for each of the five styles. Error bars represent 95% confidence intervals based on the distribution of individual MM1 values for each of the 62 participants. AD: adult-directed speech; ID: infant-directed speech. ***p < 0.001; **p < 0.01; *p < 0.05.

One way to assess the relative contribution of individual versus shared factors to preferences is to measure the amount of shared taste across participants (e.g. [33,36–38]). Here, we focused on the variability of aesthetic judgements across contrasting vocalization styles. We used a newly recorded and validated stimulus set of naturalistic but controlled a cappella vocal performances. Twenty-two female classical singers performed different melody excerpts in three contrasting singing styles—as a lullaby, as a pop song and as opera aria; and read the corresponding lyrics aloud in two contrasting ways—as if speaking to an adult audience and as if speaking to an infant. The three singing styles (that is, contrasting sounding vocalizations) were chosen as a pragmatic way to have the same singers produce contrasting performances in different styles without having to learn another specific singing technique (such as belting). The five proposed vocalization styles can be seen as a subset of possible categories of human vocalizations, sampled from a multidimensional continuum—for example, from the speech–music continuum described by Phillips [39], or from the ‘musilanguage continuum’ described by Brown [40] and extended by Leongómez *et al*. ([41], fig. 1b).

Further, the five proposed styles of vocalization allow for an interesting comparison with findings from the visual domain. Using a correlational measure of agreement (‘mean-minus-one’, MM1) and variance partitioning analysis, Vessel *et al*. [42,43] found a higher degree of shared preferences for images of faces and landscapes than for images of exterior architecture and interior architecture, and little shared taste for artworks (which reflected strong individual differences or idiosyncratic taste). They argued that naturally occurring types of stimuli, such as landscapes and human faces, have uniform behavioural relevance, which results in shared semantic meaning that is highly conserved across individuals. This would lead to similar aesthetic experience: for instance, participants tend to agree in their higher liking of an image of an oriental garden (associated with leisure) and in their lower liking of an image of a parking lot (associated with work), even when images are controlled for low-level visual features (example taken from [42]). On the other hand, artefacts of human culture, such as architecture and artwork, lack this uniform behavioural relevance and allow for the expression of individual subjects’ idiosyncratic taste. In fact, the authors suggest that this opposition between artificial (human-made) and natural (non-human-made) categories may be a fundamental organizational principle for how humans aesthetically evaluate objects [42]. If this is indeed the case, then natural types of stimuli should also elicit more shared taste in the auditory domain.

By applying this rationale to the auditory domain, and to voices in particular, and quantifying the amount of shared taste for these five types of vocalizations, this study aimed to characterize voice preferences in an integrative way while also acknowledging individual differences in these preferences. We posit that, even though all these vocalization categories are natural (in the sense that they are produced by the human vocal apparatus) and behaviourally relevant, their behavioural relevance is not uniform across individuals. Drawing a parallel with the visual domain, we argue that lullabies constitute a more ‘natural’ (in the sense of universal) kind of singing than the pop and operatic styles; and thus predicted more shared taste (that is, that participants would agree more in terms of which voices they prefer) for lullabies than for both other styles of singing. The rationale behind this was based on findings that lullabies are ubiquitous and cross-culturally recognizable [17,44,45] and arguably evolutionarily important (e.g. [46,47]), hence a more ‘natural’ (and universal) kind of singing than the pop and operatic styles. On the other hand, we expected to find more idiosyncratic taste for operatic singing, as the least ‘natural’ kind of singing of the three (that is, related to a very specific technique, and appreciated by a very specific audience). Concerning the two speaking styles, we argue that adult- and infant-directed speech are both ‘natural’ and highly behaviourally relevant in their own way. We thus expected to observe equivalent amounts of shared taste for both speaking styles.

### 1.1. Study aims and significance statement

This study aimed to empirically investigate and characterize voice preferences across a varied but controlled stimulus set of contrasting vocalizations. Given the scarcity of previous empirical research on singing voice preferences, our approach was partially and inevitably exploratory. We based our theoretical framework in parallel with the visual domain, where pioneering work has been done, to inform our predictions (table 1).

**Table 1.** Registered report design planner.

question	hypothesis	sampling plan	analysis plan	rationale for deciding the sensitivity of the test for confirming or disconfirming the hypothesis	interpretation given different outcomes	theory that could be shown wrong by the outcomes	actual outcome
1) is there a difference in the amount of shared taste across contrasting vocalization styles? (see §1.2.1).	H1A: there will be more shared taste for lullaby than for pop; and for pop than for operatic singing (MM1 lullaby > pop > opera).	60 participants rating each stimulus in terms of liking.	comparison of agreement (MM1 measures) between the three singing styles with three pairwise comparisons (paired *t*-tests, one-tailed).	sample size determined by power analysis assuming a SESOI of d*z* = 0.5, power of 0.95 and adjusting alpha for three comparisons (please see §1.3.1 for details).	only if all three planned (and directional) pairwise comparisons are significant, the results will support our hypothesis of higher shared taste for more ‘natural’/ universal (lullabies) than for more ‘artificial’ (operatic) kinds of singing, with pop in an intermediary position.	outcomes would not falsify an established theory but would suggest that predictions grounded on findings in the visual domain do (not) generalize to the auditory one, namely for vocalizations.	MM1 lullaby~opera > pop (prediction not supported).
	H1B: there will be equivalent amounts of shared taste for adult- and infant -directed speech (MM1 AD = ID)		equivalence testing of agreement (MM1 measures) between the two speaking styles to assess if the effect is statistically equivalent to zero (|effect size or d*z*| <0.5).	the sample size of 60 participants is enough for power = 0.99 in the equivalence test with equivalence bound around our SESOI of d*z* = 0.5 (or 0.1 in raw MM1 values) (please see §1.3.1 for details).	if the equivalence test is significant (and the null hypothesis test is not), the result will support that there is no meaningful difference in agreement between both styles. If both the NHST and the equivalence test are not significant, the result will be inconclusive.		MM1 AD < ID (prediction not supported).
2) on average, will the same performers be preferred across styles? (see §1.2.2)	H2: preferred performers will differ across vocalization styles.	60 participants rating each stimulus in terms of liking	based on mean liking ratings for vocalizations by each performer in each style, we will use MM1 also as a measure of interstyle agreement: if preferences are highly consistent across styles, interstyle agreement should be high (see §1.3.2).	we adopt a threshold of 0.8 (including the confidence interval) to consider preferences highly consistent across styles.	we consider interstyle agreement high (i.e., preferences highly consistent across styles) if the lower bound of the confidence interval of interstyle agreement is equal to or higher than 0.8	the finding that preferences are not highly consistent across styles (i.e., that different voices are preferred for different styles) would contradict the idea that singing and speaking voice work as ‘backup’ signals, conveying the same information about a person’s physical fitness.	interstyle agreement = 0.52 (prediction supported).

### 1.2. Hypotheses

#### 1.2.1. Hypothesis regarding the amount of shared taste across styles (question 1)

Does interrater agreement (as measured by ‘mean-minus-one’, MM1; see §2 for details) in liking ratings vary depending on the type of vocalization? Concerning the singing performances (hypothesis 1A), expanding on Vessel and colleagues’ [38,42,43] findings in the visual domain, we expected a higher degree of shared taste (higher interrater agreement) for aesthetic ratings of lullabies, a more ‘natural’ kind of singing; and lower shared taste for ratings of operatic singing (a more technical and specific type of singing), with intermediary values for pop singing. Concerning the speech performances, since both adult- and infant-directed speech seem highly behaviourally important and ‘natural’, we predicted equal amounts of shared taste for both of them.

#### 1.2.2. Hypothesis regarding average preferences for some singers (question 2)

If some voices are ‘fundamentally’ more likeable, this should happen consistently across styles, that is, the same singers/speakers should be liked the most (or the least) across styles. That is to say, if sexual selection accounts of voice attractiveness—suggesting that singing and speaking voice work as ‘backup’ signals, displaying the same (i.e., redundant) information about an individual’s physical fitness—are correct, then the rankings of favourite voices should be the same across all styles, with the ‘best’ voices consistently preferred. On the other hand, differences in preferences for singers across styles would suggest that some performers and/or voice qualities were more adequate or conformant to some styles than to others, that is, that style-specific influences are determinant of voice preferences.

### 1.3. Analysis plan and sample size justification

Our dependent variable is the participants' liking rating. Our independent variable corresponds to vocalization style (with five levels). Note that we use the terms liking and aesthetic preferences in interchangeable ways; we interpret higher liking ratings for a certain singer as indication that she was ‘preferred’, even though we do not have an explicit pairwise comparison design. Whenever requirements were met, we used parametric tests, aiming at higher power (otherwise adjusting to nonparametric alternatives; these changes are specified in our proposed analyses code).

#### 1.3.1. Comparing the amount of shared taste across styles

To test hypothesis 1A, we proposed to compare MM1 measures between the three singing styles with three pairwise comparisons (paired *t*-tests, one-tailed, adjusting *p*-values for multiple comparisons with the Holm method; or using Wilcoxon test if a nonparametric alternative was necessary, which did not happen). We expected MM1 values to be higher for lullabies than for pop performances, and higher for pop than for operatic performances (and, logically, also higher for lullabies than for operatic singing). Concerning the speech performances, we expected to find the same amount of agreement in adult- and infant-directed performances. To test hypothesis 1B, we proposed to run equivalence testing of MM1 values for these two speech styles using the two one-sided tests (TOST) procedure [48,49].

Power analysis was informed by data from previous experiments. First, to illustrate how much MM1 values vary across contrasting categories in the visual domain, Vessel *et al*. [42] reported MM1 values of 0.31 (s.d. = 0.17) for (images of) artwork, 0.38 (s.d. = 0.18) for exterior architecture, 0.40 (s.d. = 0.12) for interior architecture, 0.6 (s.d. = 0.15) for landscapes and 0.85 (s.d. = 0.12) for faces. Note that we recalculated these MM1 and s.d. estimates ourselves based on their openly available data since the original paper reports confidence intervals instead of s.d. Second, we calculated MM1 for the liking ratings of two pop melodies described in Bruder *et al*. [31] (electronic supplementary material, figure S1). In the first experiment, where participants were tested online, MM1 was 0.46 (s.d. = 0.22) for 146 ‘consistent’ participants (with test–retest Pearson correlation scores equal or superior to 0.5 in a subset of 16 repeated trials). In experiment 2, where 42 participants were tested in the lab, MM1 was 0.42 (s.d. = 0.17). We thus used an intermediary MM1 value of 0.44 (s.d. = 0.2) as a reference for our calculations. To estimate our sample size, we first stipulated the minimum difference in MM1 values necessary to statistically detect when comparing styles, i.e., our smallest effect size of interest (SESOI—e.g. [50]). We stipulated our SESOI to be a 0.1 difference in overall MM1 values per style. This corresponds to an effect size of d*z* = 0.5 (calculated using the esc_mean_sd function from the *esc* R package [51] and the values: mean group 1 = 0.44, mean group 2 = 0.34, s.d. group 1 = 0.2, s.d. group 2 = 0.2, correlation for within-subject designs = 0.5; see accompanying R script for power analyses). Concerning the correlations between repeated measures, we set it to 0.5 as a conservative estimate, since we have no grounded indication of a more appropriate value to use. But note that studies with reaction times and rating scales reportedly have high intercorrelations between the levels of a repeated-measures factor [52], and it does make sense to expect participants to be consistently more or less ‘generous’ in their use of the rating scale for liking (for instance, in the mentioned previous study, participants scoring higher on the personality trait Agreeableness systematically gave higher liking ratings [31]).

To test hypothesis 1A, the power analysis showed that, considering our SESOI of *d* = 0.5 and *α* of 0.017 (adjusted for three comparisons), we would reach power = 0.95 with a sample size of 60 participants (paired, two-sided *t*-tests, calculated with the pwr.t.test function from the pwr R package [53]). Note that resorting to the nonparametric alternative of Wilcoxon tests would not lead to great loss of power. Using the MKpower function from the MKpower R package [54], we estimated that, based on a sample size of 60 participants, we would have a power of approx. 0.9 to detect the difference in MM1 values mentioned above (i.e., the difference between 0.34 and 0.44, with s.d. = 0.2, and stipulating the same conservative *α* of 0.017). To test hypothesis 1B, the sample size of 60 participants ensures very high power for the equivalence test. Using the power_t_TOST function from the TOST package [48,49] and setting the equivalence bound between −0.1 and 0.1 (based on our SESOI in raw MM1 values), with s.d. of 0.2 and conventional alpha of 0.05, a sample size of 60 participants would ensure power of 0.99. Please see the accompanying R file for code for the power analysis. Please refer to §2.4.1 for details on how to compute MM1 and please see the electronic supplementary material, figure S2, for an illustration of this analysis conducted on simulated datasets with increasing amounts of interrater agreement.

#### 1.3.2. Assessing the consistency of average preferences across styles

For question 2, analyses are based on mean liking ratings across all participants and pooling (averaging) values of the two testing sessions. We computed a grand average of liking ratings for each singer in each vocalization style. If the same voices were preferred consistently across all styles, all pairwise correlations between styles should be high. We proposed to also use MM1 to measure this agreement across the five styles (hereafter referred to as ‘interstyle agreement’). A threshold was set at 0.8 (including 95% confidence intervals; see §2 for details), in which case average preferences would be considered highly consistent across styles. Alternatively, an interstyle agreement value (including the confidence interval) inferior to 0.8 would indicate that preferences were not highly consistent (i.e. preferences varied depending on the style). The rationale for choosing the value of 0.8 as our threshold was based on a general recommendation of this value as a minimally acceptable level of reliability [55] and on our own data simulations, which allowed us to observe that this value indeed corresponds to a high level of consistency in preferences across styles. Please see §2.4.2 for details on how to compute interstyle agreement and please see the electronic supplementary material, figures S3 and S4, for a simulation-based demonstration of this solution. Note that this is a descriptive approach that does not fit into conventional hypothesis testing based on *p*-values, nor does it allow for power analysis. However, it did allow us to test our prediction: we would conclude that preferences were highly consistent if the lower bound of the confidence interval was equal or superior to 0.8.

## 2. Method

### 2.1. Participants

Participants (30 self-reported as female, 32 as male; aged between 22 and 85 years, *M* = 46 years, s.d. = 19) were recruited from the participant database of the Max Planck Institute for Empirical Aesthetics, in Frankfurt, Germany, which consists of adults, mostly lay listeners, with a preponderance of students and retired persons. While we acknowledge that this convenience sample shares the generalizability limitations of most studies sampling from ‘WEIRD’ populations (White, Educated, Industrialized, Rich, and Democratic [56]), we attempted to enhance the representativity of the sample by examining participants with a large range of musical expertise (i.e. not recruiting only musically trained participants) and keeping balanced genders in the recruited sample. Participants were rewarded for their participation at a rate of 7€ per half hour. The only exclusion criterion for participation in data collection was reported hearing impairments. Note that we collected data from 62 participants instead of the 60 participants planned based on the power analysis (§1.3.1) to ensure timely completion of data collection in case of missed testing sessions and/or data exclusions. We proposed to exclude from analyses data from participants whose scores were the same for more than 85% of trials. This was specified in our analysis code, but, ultimately, no participants had to be excluded. The experimental procedure was ethically approved by the Ethics Council of the Max Planck Society (no. 2017_12) and was undertaken with written informed consent of each participant.

### 2.2. Materials

#### 2.2.1. Questionnaires for collection of participant-related data

In the end of the first testing session, participants were asked for the following information, to be used in exploratory analyses:

- demographic questions about age, mother tongue, gender (female/male/non-binary/prefer not to disclose/prefer to self-describe), and sexual orientation (heterosexual or straight/gay or lesbian/bisexual/prefer not to disclose/prefer to self-describe).
- questions about their experience while doing the experimental task : (i) Did you perform the task conscientiously? (ii) Did you recognize the language spoken and sung in the stimuli (if yes, which was it)? (iii) Do you have any comments about your experience while doing the task? (iv) During the experiment, each block of trials contained different types of vocalization. How would you label the five types of vocalization you listened to?
- the 18-item version of the general Music Sophistication subscale from the Goldsmiths Music Sophistication Index (Gold-MSI [57]), as computed with the Gold-MSI configurator (https://shiny.gold-msi.org/gmsiconfigurator). The Gold-MSI is a self-report measurement instrument to assess musical skills and behaviours in the general population.
- a short questionnaire about music preferences, asking participants how much they liked or disliked certain general music genres while trying to distinguish if they simply did not like something or if they really disliked it. We used only four of the genres investigated by Siebrasse & Wald-Fuhrmann [58]: pop, opera, rock and non-European music.

#### 2.2.2. Stimulus set

The stimuli proposed for this study come from a newly recorded stimulus set comprising singing and speech performances. Detailed information about the singing performances is presented in Bruder & Larrouy-Maestri [59]. In what follows, we summarize the findings that are relevant to the current study. The stimulus set consists of vocalization by 22 highly trained Brazilian female classical singers (16 sopranos, 6 mezzo-sopranos, aged from 22 to 45 years old, *M* = 32.5, s.d. = 7.1), with vocal training ranging from 4.5 to 27 years (*M* = 12.9 years, s.d. = 6). Singers were recorded in a professional music recording studio in Sao Paulo, Brazil, and performed the same melody excerpts (the first phrase of different songs) as a lullaby, as a pop song, or as an opera aria, and spoke the corresponding lyrics aloud as if directed to an adult audience and as if directed to an infant. Singing stimuli are on average 9 s long, and speech stimuli are on average 5 s long. The exact instructions given to singers during the recording session were as follows. For lullaby singing, imagine you have a baby on your chest and you want to make it sleep. For pop singing, imagine you are performing a pop song with a microphone. For operatic singing, imagine you are on stage performing an opera aria. For adult-directed speech, imagine you are reading out loud the translation of the lyrics from something you have just performed on stage. For infant-directed speech, read the same text out loud but this time imagine you are talking to a baby or a small child. Operatic singing was performed with higher pitch than pop and lullaby (one fourth or one fifth higher, depending on the range of the melody), aiming at naturalistic performances and considering that operatic singing typically has higher pitch than both other styles. The singing stimuli were validated in lab experiments (two forced-choice tasks, *n* = 25 participants per stimulus or higher) where participants were asked to indicate, in each trial, if a given singing performance sounded like a lullaby, a pop song or an opera aria; and if a given speech performance was directed to an adult or a baby/child. For the subset of stimuli to be used in the current study, the proportion of correct recognition was higher than 67% for all styles. The proposed subset consists of three melody excerpts: ‘Nana Nenê’, originally a lullaby; ‘Chove Chuva’, originally a MPB (Música Popular Brasileira, a genre of Brazilian popular music) song by Brazilian artist Jorge Ben Jor (1939–) and Melodia Sentimental, originally an art song by Brazilian classical composer Heitor Villa-Lobos (1887−1959). This leads to 330 performances (22 singers performing three melody excerpts in five vocalization styles). We chose to use performances with lyrics in Brazilian Portuguese (a version of each performance with /lu/ sound is also available in the dataset) to preserve the phonetic variability of speech. For the present study, we loudness normalized all stimuli to −23 LUFS, thus controlling for possible influences of loudness in liking ratings. Please see the electronic supplementary material, figure S5, for sheet music of the melody excerpts. The stimuli used in the present work are currently available at https://osf.io/8k4af.

### 2.3. Procedure

The experimental session was as follows: after general instructions, the experiment started with three training trials to familiarize participants with the task. Participants were asked to rate how much they liked each stimulus on a scale of 1 (*not at all*) to 9 (*a lot*), by clicking with the mouse on the corresponding number on the scale presented on the computer screen. In each trial, a ‘Next’ button became visible only after the stimulus ended. Clicking on this button prompted the next trial and playing of the next stimulus. Each stimulus was played only once. The experiment was divided into five blocks, one for each style of vocalization. Each block comprised 66 trials, corresponding to one performance by each of the 22 singers for each of the three melodies, presented in a randomized order. The order of these blocks was counterbalanced across participants. Participants completed the experiment at their own pace and needed between 58 and 90 min for the first session, and between 48 and 70 min for the second session. Breaks were proposed between blocks. At the end of the experiment, participants were asked to complete the questionnaires mentioned above. Participants completed two testing sessions (test–retest), no longer than 14 days apart from each other, and preferably one week apart. The rationale behind stipulation of this time interval is as follows. According to Allen & Yen [60], two aspects need to be considered when testing reliability with the test–retest method: the possibility of learning, carry-over or recall effects (i.e., that the first testing may influence the second); and the possibility of a change in status of the measured trait between sessions (e.g., change in a cognitive ability in children). None of these aspects was of particular concern in our paradigm. Given the high number of stimuli (330), the possibility of participants remembering their answers from one session to the next was probably negligible, and music abilities and engagement seem to be relatively stable among adults. Müllensiefen *et al*. [57] report a test–retest correlation of *r* = 0.86 or higher for all subscales of the Gold-MSI self-report inventory, with participants tested on average 23 days apart (s.d. = 9.2); and George & Ilavarasu [61] report test–retest reliability of *r* = 0.87 for a 15-day interval and *r* = 0.91 for a 1-month interval in the validation of their Music Receptivity Scale. We thus privileged pragmatic aspects of data collection in our decision to propose the test–retest interval. The second session was identical to the first one, with the exception that no questionnaires had to be filled. Stimuli were presented and data were recorded in the experimental platform Labvanced [62]. The whole experiment was conducted in German. Participants were tested in the laboratories of the Max Planck Institute for Empirical Aesthetics, in Frankfurt, Germany.

### 2.4. Data analyses

All analyses were performed using R Statistical Software [63] and R Studio [64]. Please see the accompanying .Rmd scripts for code to run all of the proposed analyses.

#### 2.4.1. Shared taste or interrater agreement

We measured interrater agreement (or shared taste) by computing the ‘mean-minus-one’ (MM1) measure, a leave-one-out type of correlational agreement measure [42,43]. To compute MM1, a Pearson correlation is computed between a given participant’s liking ratings for the stimulus set and the average ratings of all other participants. This is done for all participants in the sample. The resulting individual correlations are then converted to *z*-scores (Fisher’s *r*-to-*z* transform), averaged and converted back into an *r* score (*z*-to-*r* transform) for easier interpretation of the final MM1 measure. This method has been shown to result in less biased estimates than averaging raw correlations [65].

#### 2.4.2. Consistency of preferences for some singers (interstyle agreement)

As outlined in our analysis plan, we proposed to use MM1 also to measure interstyle agreement, that is, to assess how consistent were average preferences for some singers across the different styles of vocalization. Based on grand averages of liking ratings for each singer in each vocalization style, we computed interstyle agreement using the same code used to compute MM1 interrater agreement, but with the five styles as the ‘raters’ who ‘judge’ the 22 singers. That is, a Pearson correlation was computed between a given style’s (grand average) ratings of singers and the average of the (grand average) ratings of the four other styles; the same was done for all styles; the resulting five individual correlations were converted to *z*-scores, averaged, and converted back into an *r* score, which is our ‘interstyle MM1’, used to assess how much styles ‘agree’ with each other. Interstyle agreement is considered high if it is equal or superior to 0.8 (including its 95% confidence interval).

#### 2.4.3. Exploratory analyses

Our design with two testing sessions allowed us to conduct complementary analyses: we assessed intrarater agreement and computed the beholder index [33]. Additionally, to contribute to methodological discussions about agreement measures (see [34,66]), we also report the more widely known measures of Kendall’s coefficient of concordance (more commonly used in the voice attractiveness literature), Krippendorff’s *α* and Intraclass Correlations (ICC). For completeness, we report these two last measures both for interrater agreement in question 1 and for interstyle agreement in question 2.

#### Intrarater reliability

For each participant, Pearson correlation scores were computed based on the ratings of the 330 stimuli in the first and the second sessions, as a measure of test–retest intrarater reliability. Measuring how self-consistent participants are is vital to understand how much participants can agree with each other. If the interrater agreement is low but the intrarater consistency is high (i.e. participants’ ratings are consistent between test and retest), one can be confident that ratings were not random, but instead indicate a preponderance of private or idiosyncratic taste. A similar pattern was reported by Bruder *et al*. [31], where test–retest reliability was high for about half of the online participants (*r*_test–retest_ ≥ 0.5) but very low interrater agreement indicated highly idiosyncratic preferences for pop singing.

#### Variance component analysis and beholder index

While we focus our hypothesis testing on MM1, we also report and jointly discuss the beholder index [33] as a complementary measure of agreement. Based on generalizability theory [67], Hönekopp [33] proposed the beholder index as a measure of the amount of private taste in ratings of attractiveness of face stimuli. To estimate the beholder index (‘bi’), one needs at least two sets of ratings by each rater. Variance components are computed and bi is estimated as a ratio between the amount of private taste and the total meaningful (i.e. accounted for or non-residual) variance. Beholder index estimates should thus mirror MM1 estimates (i.e. when MM1 is high, bi should be low and vice-versa). To estimate bi, one first needs to conduct a variance component analysis (VCA). One convenient way of conducting VCA is to compute a multilevel model (using the lmer function from the lme4 package in R [68]) with random intercepts for stimuli, participants, blocks and all two-way interactions between these terms [66]. VCA allows one to compare the variance in different clusters, which are components that are similar across measurements, such as raters, stimuli or occasions, and are treated as if they are sampled from a random population [66]. In this context, the variance in the stimulus cluster is related to shared taste; the variance in the rater × stimulus cluster is related to idiosyncratic or private taste (and would allow inferences about differences in ranking preferences across participants); and the interpretation of the rater cluster is controversial: while it seems to be related to individual differences (e.g. personality, mood), it is not clear whether it should count as a source of idiosyncratic contribution for judgement [33]. For example, if ratings of three stimuli by multiple raters lead to average ratings of 3, 4 and 5, and one particular rater gives out the ratings of 1, 2 and 3, respectively: this two-point difference compared with the average ratings may be interpreted as meaningless differences in scale use, since they are in the same direction as the average ratings, thus indicating agreement with the average ratings and with the overall ranking of stimuli. Alternatively, this difference may reflect genuine differences in perception, in the sense that this rater disagrees with the average taste (thus indicating private taste). Hönekopp [33] proposes two different versions of the beholder index: bi_1_, that disregards the rater cluster, and bi_2_, that takes the rater cluster into account. Please refer to [33, p. 2] for formulae; to our accompanying R files for code to compute these indices; and to the electronic supplementary material for an example of these analyses conducted on previous data from [31] (electronic supplementary material, figure S6). Note that, once data collection was complete, we tried including a singer cluster to VCA, but that led to models with convergence issues. We ultimately opted to keep a simpler model structure of lmer (liking_rating ~ 1 + (1 | rater) + (1 | item) + (1 | time) + (1 | rater : item) + (1 | rater : time), d*f*). Finally, we eliminated the time and item interaction (1 | time : item) because that cluster captured no variance. Also note that since there is no straightforward way to summarize the precision of variance components [69], and bi is a ratio between variance components, comparisons between estimated bi across conditions must remain descriptive, i.e. no confidence intervals or standard deviations can be reported. This is why we focused on MM1 measures to test hypothesis 1, though we hoped to gain considerable insight into participants’ preferences based on bi estimates.

#### Krippendorff’s α

To allow for direct comparison with other studies, we also report Krippendorff’s *α* as an alternative measure of interrater agreement. Krippendorff’s *α* is a generalization of several known reliability indices, and widely applicable [55,70]. We used the kripp.boot function from the kripp.boot R package [71], which implements Krippendorff’s algorithm [70] for bootstrapping the *α_K_* coefficient and 95% confidence intervals. We ran 100 iterations and took the resulting mean value of all of the bootstrapped replicates. Note that in our simulations, this value was close to the output of the kripp.alpha function from the irr R package [72]; and 10 or 100 iterations produced very similar results.

#### Intraclass correlations

A more widely known interrater agreement measure, ICC, is also reported to allow for comparison with other studies. We used the ICC function in the psych R package [73] to compute ICC2 (single random raters, absolute values).

#### Kendall’s coefficient of concordance

To allow for comparison with other studies, we also report Kendall’s coefficient of concordance, *W* [74,75], a nonparametric statistical test. *W* is proportional to the average rank-order correlation among all pairs of raters. We used the Kendall function from the irr R package [72].

## 3. Results

### 3.1. Question 1: shared taste or interrater agreement

Figure 1*a* displays the overall pattern of liking ratings by style of vocalizations in the first and second testing sessions, and figure 1*b* displays interrater agreement, as measured by MM1. The planned comparisons revealed that both predictions from hypothesis 1 were not supported: for the singing performances, MM1 was indeed higher for lullaby (MM1 = 0.51, 95% CI [0.46, 0.55]; CI computed based on *z*-transformed values and transformed back to *r*-scores for easier interpretation) than pop singing (MM1 = 0.40, 95% CI = [0.35, 0.44]; *t*(62) = 5.43, *p* < 0.001), but it did not differ from operatic singing (MM1 = 0.49, 95% CI = [0.44, 0.54]; *t*(62) = 0.69, *p* = 0.247). In fact, equivalence testing showed that, for our specified SESOI of *d*_Z_ = 0.5, these values are equivalent: *t*(61) = −2.84, *p* < 0.01; and pop and operatic singing did differ from each other, but not in the expected direction (i.e., MM1 was higher for operatic than pop singing; *t*(62) = 3.73, *p* < 0.001). For the speech performances, MM1 was higher for infant-directed speech (MM1 = 0.54, 95% CI [0.50, 0.59]) than adult-directed speech (MM1 = 0.36, 95% CI [0.32, 0.40]; *t*(61) = −7.06, *p* < 0.001).

### 3.2. Question 2: consistency of preferences for some singers (interstyle agreement)

**Figure 2.**
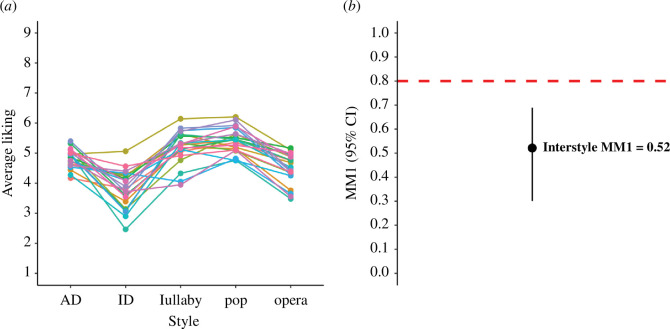
Interstyle agreement. (*a*) Ranking of singers based on average ratings per singer by style. Each colored line corresponds to one singer. (*b*) Interstyle agreement. Error bars represent 95% confidence intervals based on the distribution of individual MM1 values calculated for the five styles. The red dashed line indicates the specified threshold of 0.8, above which preferences for certain singers would be considered highly consistent. AD: adult-directed speech; ID: infant-directed speech.

The visual inspection of the ranking of singers (figure 2*a*) reveals some consistency in singer preferences across styles, which is supported by the computed value of interstyle agreement of 0.52 (95% CI [0.30; 0.69]; computed over *z*-scores and transformed back to *r*-scores for ease of understanding). However, according to our specified threshold of 0.8 (figure 2*b*), this value is not considered highly consistent, which confirms our prediction for hypothesis 2.

### 3.3. Exploratory analyses

#### 3.3.1. Distribution of liking ratings across styles

Liking ratings (figure 1*a*) differed for each of the styles in pairwise comparisons with all other styles (all *p*s < 0.001; based on average ratings of sessions 1 and 2 and adjusting *p*-values for multiple comparisons with the Holm method). Liking ratings were lowest for infant-directed speech (*M* = 3.81, 95% CI [3.52, 4.09]), followed by operatic singing (*M* = 4.51, 95% CI [4.13, 4.88]), adult-directed speech (*M* = 4.83, 95% CI [4.56, 5.10]), lullaby singing (*M* = 5.20, 95% CI [4.85, 5.56]) and highest for pop singing (*M* = 5.42, 95% CI [5.13, 5.7]).

#### 3.3.2. Correlations between average liking by singer across pairs of styles

When computing interstyle agreement, we wondered if the average singer preferences depicted in figure 2*a* were more similar across some pairs of styles than others. The electronic supplementary material, table S1, presents an overview of Pearson correlations between average liking ratings by singer across pairs of styles. The correlation between average liking by singers in adult- and infant-directed speech was −0.07. Correlations between adult-directed speech and any of the singing styles were all weak (between 0.2 and 0.38), as were correlations between infant-directed speech and any of the singing styles (between 0.16 and 0.33). Finally, correlations involving singing styles were between moderate and strong (between 0.57 and 0.71). These results suggest that, even though there is some consistency in average preferences for certain voices across styles, this consistency seems to be limited to singing styles and is not generalizable to all vocal performances. Note that the same pattern is observed when subsetting only male heterosexual participants (*N* = 29) in our sample: the correlation between adult-directed speech and pop singing is then *r*(20) = 0.37 (*p* = 0.087); and *r*(20) = 0.37 (*p* = 0.089) when grouping all speech and all singing performances into two domains.

#### 3.3.3. Intrarater reliability

The average test–retest intrarater reliability score was 0.49 (s.d. = 0.16, 95% CI [0.45, 0.53], range: between −0.13 and 0.78), which is similar to the values reported by Bruder *et al*. [31] for pop singing performances (average *r*_test–retest_ = 0.4, s.d. = 0.29, 95% CI [0.37, 0.43]), supporting the idea that participants’ preferences are generally consistent over time, albeit with sizeable individual differences. Please see the electronic supplementary material, figure S7, for a histogram of individual *r*_test–retest_ scores.

#### 3.3.4. Variance component analysis and beholder index

For both versions of the beholder index, we observed high proportions of private taste: bi_1_ was 0.59 for infant-directed speech, 0.61 for lullaby singing, 0.61 for operatic singing, 0.74 for pop singing and 0.75 for adult-directed speech. These values indicate that, from the variance that is time-stable and accounted for (or not residual), between 59% and 75% corresponded to private taste, depending on the vocalization style. In other words, only between 41% and 25% was attributable to shared taste. These numbers are even more extreme for bi_2_, which considers the variance in the rater cluster as contributing to private taste: bi_2_ was 0.80 for infant-directed speech, 0.87 for lullaby singing, 0.89 for operatic singing, 0.89 for pop singing and 0.90 for adult-directed speech (or, conversely, only between 10% and 20% of the meaningful time-stable variance was attributable to shared taste). Though both versions of the beholder index led to similar inferences as MM1, bi_1_ mirrored MM1 slightly better than bi_2_ (upon visual inspection—no formal tests were conducted). Please see electronic supplementary material, figure S8, for the results of the supporting VCA, and electronic supplementary material, figure S9, for the two versions of the beholder index across the five styles of vocalization.

#### 3.3.5. Alternative interrater agreement measures

**Figure 3.**
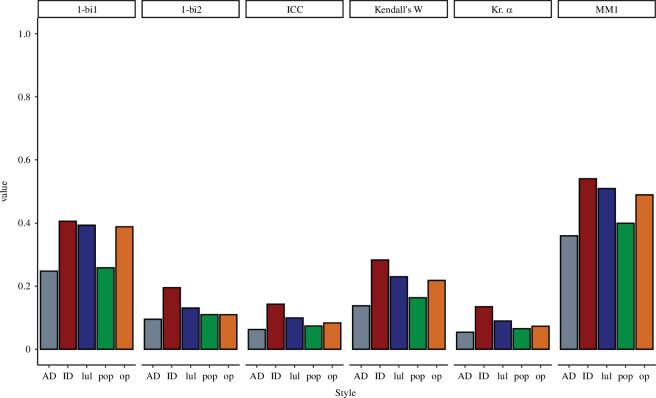
Proposed measures of interrater agreement for every style. bi_1_ and bi_2_: beholder index in versions 1 and 2, respectively. ICC: intraclass correlations or ICC[1,2] (absolute agreement, single random raters); Kendall’s *W*: Kendall’s coefficient of concordance; Kr. *α*: Krippendorff’s *α*; MM1: ‘mean-minus-one’ agreement; AD: adult-directed speech; ID: infant-directed speech. For all measures, perfect agreement would correspond to a value of 1.

All interrater agreement measures supported similar inferences about the data, albeit with different magnitudes of agreement (figure 3). As anticipated, Krippendorff’s *α* and ICC [2,1], as measures of absolute agreement, led to similarly low values, but the relationship between the amount of agreement across styles was roughly well preserved when compared with the pattern obtained with MM1. Interrater agreement was highest for infant-directed speech, followed by lullaby, operatic and pop singing, and was lowest for adult-directed speech—though note that we did not conduct any statistical comparisons between these values. Kendall’s coefficient of concordance, a nonparametric correlational measure of agreement, approximated MM1 values a little more closely. For easier comparison, in figure 3, we present 1-bi_1_ and 1-bi_2_ as measures of shared taste (since private and shared taste should add to 1). Note that we did not include Cronbach’s *α* because it is an inadequate measure of interrater agreement [33,34], but just for comparison, Cronbach’s *α* for our data would be 0.95 for lullaby singing and infant-directed speech, 0.94 for operatic singing, 0.91 for pop singing, and 0.90 for adult-directed speech.

#### 3.3.6. Alternative interstyle agreement measures

Alternative measures of ‘interstyle Krippendorff’s *α*’ and ‘interstyle ICC’ were also proposed for completeness. Once again, these measures of absolute agreement led to much lower values than ‘interstyle MM1’. ‘Interstyle Krippendorff’s *α*’ was 0.004 (bootstrapped 95% CI [−0.172, 0.149]), and ‘interstyle ICC’ was 0.12. Note that in the simulations reported earlier (electronic supplementary material, figures S3 and S4), we compared these measures, and also found that ‘interstyle Krippendorff’s *α*’ and ‘interstyle ICC’ consistently led to lower results than interstyle MM1. The difference between ‘interstyle MM1’ and the other two measures seems to be even more prominent with the experimental (and more complex) data reported here. Considering the present research question of average preferences, we would argue that ‘interstyle MM1’ is indeed the most adequate measure from all three, while ‘interstyle Krippendorff’s *α*’ and ‘interstyle ICC’, as measures of absolute agreement, are probably too strict to properly represent the trends we are interested in here.

#### 3.3.7. Participant characteristics

##### General music sophistication and MM1 agreement

Participants’ average music sophistication score according to the Gold-MSI subscale for general music sophistication was 76.9 (s.d. = 24.6). For reference, the normative value reported in the original study by Müllensiefen *et al*. [57] was 81.6 (s.d. = 20.6). We explored the relationship between participants’ general music sophistication, as estimated by the Gold-MSI, and how much they agreed with each other (separately for each vocalization style, and based on individual MM1 scores for each style). In line with previous findings that more musically sophisticated participants tend to agree more with the group evaluation of pop singing performances [31], there were positive correlations between participants’ general music sophistication scores and their MM1 scores in all three singing styles (opera: *r*(60) = 0.40; pop: *r*(60) = 0.33; lullaby: *r*(60) = 0.40; correlations computed based on *z*-transformed values of MM1). However, there was no correlation between participants’ general music sophistication scores and their MM1 scores in adult-directed speech (*r*(60) = −0.02) or infant-directed speech (*r*(60) = −0.06). That is, more musically sophisticated participants tended to agree more with the group aesthetic evaluation for the three categories of singing, but not for the two categories of speech. Please see electronic supplementary material, figure S10, for an illustration of these relationships.

##### Opera fans

We also explored the relationship between participants’ musical taste, as measured with a musical preferences questionnaire, and participants’ individual MM1 scores for each style. Particularly, we wondered if the higher than expected MM1 for operatic singing could be related to a prevalence of opera fans in our sample. Inspection of the results of the musical preferences questionnaire (electronic supplementary material, table S2) shows that 31 participants indicated liking opera as a musical genre (14 answered ‘I like it a bit’ and 17 ‘I like it a lot’), 15 indicated disliking it (5 answered ‘I don't like it at all’ and 10 ‘I rather dislike it’), and 16 could be considered neutral or unfamiliar with the genre (3 answered ‘I don’t know/hardly know’ and 12 answered ‘neutral’; note that one participant’s questionnaire responses were not recorded due to a technical error). Participants were thus grouped into ‘opera fans’, ‘opera nonfans’ and ‘neutral/unfamiliar.’ Please see electronic supplementary material, figure S11, for illustrations of these comparisons. Although individual MM1 values seemed to be higher for opera fans, pairwise comparisons between individual MM1 scores of these three groups did not reach statistical significance (opera fans: *M* = 0.51, s.d. = 0.18; opera non-fans: 0.46, s.d. = 0.15; neutral/unfamiliar: *M* = 0.39, s.d. = 0.24; all *p*s > 0.28; two-tailed *t*-tests based on individual *z*-transformed values of MM1, correcting for multiple comparisons with the Holm method). Note that we were underpowered to reliably demonstrate these differences in such small groups of participants, but Cohen’s *d* for the comparison between opera fans and neutral participants was 0.55, which corresponds to a medium effect size; it was 0.36 for the comparison between opera fans and non-fans, and 0.27 (small effect size) for the comparison between non-fans and neutral participants. Further experiments with larger samples would be necessary to clarify these trends.

##### Pop fans

We conducted the same exploratory analysis for MM1 agreement for pop performances in relation to participants’ declared musical preferences for the pop musical genre. According to the musical preferences questionnaire, 47 participants liked pop music, 11 were neutral and 4 did not like it, leading to very different group sizes of popfans and pop nonfans. There were no significant or meaningful differences between groups in terms of their individual MM1 values for pop performances (pop fans: *M* = 0.39, s.d. = 0.17; neutral participants: *M* = 0.37, s.d. = 0.18; pop non-fans: *M* = 0.38, s.d. = 0.11; all effect sizes < 0.11).

## 4. Discussion

We explored preferences for the human voice with an integrative approach, comparing the amount of shared taste or interrater agreement across contrasting categories of vocalizations. We found highly idiosyncratic taste for all vocalization styles. Considering that lullabies are a more ‘natural’/universal category of singing (with arguably more uniform behavioural relevance across listeners), we predicted higher shared taste for lullabies than pop; and lower shared taste for operatic singing, which seems to be a more ‘artificial’ category of singing. However, results did not support our directional hypothesis: while the amount of shared taste was indeed higher for lullaby than for pop singing, it was higher for operatic than pop singing and equivalent to lullaby singing. At first, these findings suggest that predictions grounded on findings in the visual domain do not generalize to the auditory one, namely for vocalizations. After reflecting on our results, however, we would argue that such a conclusion may be premature and that more evidence is needed before definitively concluding that predictions grounded in the visual domain do not generalize to vocalizations.

Regarding the higher than expected shared taste for operatic singing, we would speculate that it relates to the relatively high presence of opera fans in our sample: half of the participants indicated liking opera a bit or a lot, which suggests that opera may be a specially important and circumscribed category of singing for these participants. These opera fans seemed to be more musically sophisticated and to agree more with each other in terms of their preferences than opera non-fans and ‘opera neutral’ participants in the sample, though these comparisons were exploratory and the reported differences were not statistically significant (despite moderate effect sizes). Studies indicate that expertise can modulate aesthetic preferences for visual art and dance [76,77] and music (e.g. [78–80]). When judging pitch accuracy of singing performances, laymen’s and experts’ definitions of pitch accuracy overlap, but there are differences regarding the musical criteria employed in the evaluation of voices [81,82]. That is to say, the opera fans in our sample likely had considerable expertise regarding operatic singing, which could lead to shared stylistic conceptions of what constitutes a good operatic singing performance. To disentangle the role of expertise and preferences for certain musical styles, it would be interesting to recruit larger samples of participants with widely diverse musical backgrounds and preferences. Experiments with participants from other cultures, especially ‘non-WEIRD’ [56] samples, could also provide valuable insight: we would expect to find lower shared taste for operatic singing among participants from cultures unfamiliar with opera and classical singing. On the other hand, we would expect to find higher shared taste for lullabies also cross-culturally: lullabies seem to be a uniquely stable song category, which, according to Trehub & Trainor [23], may be related to the higher stability of child care contexts across cultures, when compared with other aspects of community life. Lullabies may thus be the most universal category of singing also from the point of view of preferences—one prediction worth testing in future cross-cultural studies. The ‘pop singing category’, on the contrary, is much less circumscribed and challenging; the term ‘pop’ is very vague, and what people understand by ‘pop music’ is more likely to vary, both within and between cultures.

Regarding the amount of shared taste in speech performances, our prediction of equivalence in the amount of shared taste in the two categories was also not confirmed: there was higher shared taste for infant- than for adult-directed speech. This higher shared taste for infant-directed speech seems to be driven by a shared dislike, as indicated by the lower liking ratings for this style. Anecdotally, some participants stated ‘I found the baby talk annoying’ after their testing sessions. Considering that infants prefer infant-directed speech over adult-directed speech [83,84]; that the acoustic characteristics of infant-directed speech change as infants develop [19], and that age-specific preferences for infant-directed speech have been reported [85], it would be interesting to investigate in which point in development infant-directed speech becomes less pleasurable. Relatedly, Saxton *et al*. [86] compared voice attractiveness ratings by children, adolescents and adults and found that voice attractiveness judgements change with age, suggesting that adult-like judgements of opposite-sex voices arise at puberty. We thus wonder if children would like (or tolerate) infant-directed speech more than adults, and if adolescents would share adults’ reduced liking of it. The dynamics of voice preferences, including singing voice preferences, throughout human development present an exciting avenue for future research.

Concerning our second hypothesis, the prediction of limited consistency of average preferences for some voices across styles was supported by an interstyle agreement of 0.52. According to the specified threshold of 0.8, this is not considered highly consistent. Our findings thus contradict the idea that singing and speaking voices work as ‘backup’ signals, conveying the same information about a person’s physical fitness. The exploratory analysis of pairwise correlations between styles in average liking ratings by singers (electronic supplementary material, table S1) further challenges this idea. All pairwise correlations involving either one of the speech styles were small, the highest of them being *r* = 0.38 (between adult-directed speech and pop singing). Only correlations between pairs of singing styles were moderate to high. This suggests that musically-related factors may be driving these similarities—an interpretation also supported by the reported correlations between participants’ general music sophistication scores and individual MM1 values only in the case of the three singing styles, but not in the case of the speech styles. While our findings cannot falsify the ‘singing and speaking as backup signals’ idea, and sexual selection may still be one of many factors influencing voice preferences, the role of sexual selection in the singing voice preferences reported here appears to be limited. Importantly, our results contrast with findings reported by Valentova *et al*. [32], who recorded the same individuals singing and speaking and asked lay listeners (heterosexual participants of the opposite sex) to rate the voice attractiveness of these stimuli. They reported Pearson correlation scores of 0.67 for female voices and 0.72 for male voices between mean attractiveness ratings of singing and speaking. In our data, the Pearson correlation score between mean liking ratings of adult-directed speech and pop singing was *r*(20) = 0.38, even when subsetting only male heterosexual raters (0.05 < *p*s < 0.1). These contrasting results may be due to differences in terms of stimulus material (e.g. language, key of singing performances), rated attribute (voice attractiveness versus liking rating), recorded subjects (untrained versus highly trained singers) and listeners' cultural background. We also speculate that because Valentova *et al*. [32] recorded untrained singers and allowed them to sing in any key, their singing stimuli might be perceptually closer to the corresponding speaking performances (e.g. on the speech–music continuum [39]). In contrast, our framework likely produced singing stimuli that were more distinct from the speaking performances and were possibly more influenced by musical factors that contribute to voice preferences.

To facilitate comparisons with other studies, we report several complementary measures of interrater agreement. Similar to the findings of Kramer *et al*. [34] for social judgements of faces, we observed that different interrater agreement measures yielded consistent inferences about the data. Importantly, although the magnitude of values varied depending on the measure used, all measures indicated: (i) higher shared taste for infant-directed speech, followed by lullaby, pop and operatic singing, with the lowest shared taste for adult-directed speech, and (ii) a prevalence of private taste for all styles.

Our findings of very limited shared taste across all categories of vocalizations agree with previous research on singing voice preferences [31], but contrast with studies on speaking voice attractiveness reporting low to moderate interrater agreement. For comparison, Kendall’s concordance coefficient ranged from *W* = 0.19 to 0.62 in previous studies [24–26,87]. In our data, Kendall’s *W* was 0.14 for adult-directed speech (or 0.12 based on 29 heterosexual male raters). Though one should be careful when comparing studies that used such varied stimulus material (i.e. ranging from vowels and wordlists to counting from 1 to 10, to reading a story aloud), and experimental procedures, it seems that voice attractiveness ratings for the speaking voice typically lead to higher interrater agreement than what we report here for liking of adult-directed speech.

We suggest that future research should aim to further characterize voice preferences in a more integrative way, by measuring shared preferences for songs and vocalizations spanning different geographic, linguistic and cultural contexts, as seen in recent cross-cultural studies [15–17,21,88]. In addition to the cultural factors previously discussed, future studies could also explore a larger range of vocalizations. In this study, we only examined two styles of speech performances, and our adult-directed speech condition was closer to declamation than prose. It would be valuable to explore other regions of the speech–music continuum, such as informal prose, recitative, rap, sprechstimme and poetry. Examining a broader range of vocal styles could reveal nuanced patterns of preference and shared taste and potentially uncover new factors that contribute to voice preferences. Whenever possible, infant-directed vocalizations should be recorded in the actual presence of infants, since simulated infant-directed vocalizations are reportedly less efficient and ‘loving sounding’ than real-time performances where an infant is present [89–91].

We conclude by reflecting on the insights of Hönekopp [33] regarding the question of shared taste in face attractiveness ratings. Hönekopp [33] highlighted that prior research had focused on average ratings of face attractiveness, leading studies to present face attractiveness as a highly shared attribute. By computing the beholder index, he showed that only about half of the time-stable meaningful variance in face attractiveness ratings was shared, with considerable differences depending on how varied the stimulus set was. Similarly, we show that, no matter the agreement measure used, voice preferences are highly idiosyncratic. We thus question the emphasis of previous research on correlates of average voice attractiveness. We encourage future research to look closely into private taste and individual differences in voice preferences, as these may offer more nuanced and informative insights.

## Data Availability

Please find the analysis code (R markdown files), an electronic supplementary information file and stimuli at [92]. Please find the publicly registered Stage 1 manuscript here [93]. Supplementary material is available online [94].
